# Supplementary material for: Cryo-EM structures of the translocational binary toxin complex CDTa-bound CDTb-pore from Clostridioides difficile
Source: Nat Commun. 2022 Oct 17;13:6119. doi: 10.1038/s41467-022-33888-4 (PMC9576733; doi:10.1038/s41467-022-33888-4)
Supplement: Supplementary file 1 — Supplementary Information [file 41467_2022_33888_MOESM1_ESM.pdf]

Supplementary information

for

Cryo-EM structures of the translocational binary toxin complex CDTa-bound CDTb-pore from *Clostridioides difficile*

Akihiro Kawamoto, Tomohito Yamada, Toru Yoshida, Yusui Sato, Takayuki Kato, and Hideaki Tsuge*

***e-mail:** tsuge@cc.kyoto-su.ac.jp

Supplementary note

The structure of the anthrax toxin PA-pore has been determined in its apo form[18], and EF- and LF-bound forms[1] by cryo-EM (Supplementary Fig. 8). The atomic structures of the LF-and EF-bound forms show that the first α-helix unfolds and docks into a deep amphipathic cleft, called the α-clamp, which is located between the two Ca-edges of PA (Supplementary Fig. 8). The binding modes of EF and LF are similar, as both use the first α-helix to bind the α-clamp, and the 20–30 N-terminal residues are missing, possibly continuing to the ϕ-clamp located 35–40-Å away. The structure of the PA-pore shows a striking similarity to the CDTb-pore and Ib-pore. However, the substrate components differ in their activity, structure, and binding mode between CDTa/Ia and LF/EF. Structurally, CDTb and Ib have the α-clamp in similar regions, where two adjacent Ca-edges are created, but both CDTb and Ib do not use the region for α-helix binding. In the PA-pore, the α-clamp was proposed to be important for the non-specific binding of the α-helix and subsequent translocation. The α- and ϕ-clamps appear to operate allosterically, with peptide binding at the α-clamp site being required for the allosteric gating of the ϕ-clamp to a clamped state[2]. In the CDTb-pore and Ib-pore, an α-clamp is not needed for binding; instead, these pores use a different way to unfold the N-terminal α-helix. Whether the conformation of the ϕ-clamp is flexible or the secondary structure of the substrate unfolds during the translocation remains unclear. The ϕ-clamp structures seem stable in the same configuration as closed (6-Å diameter) in all reported structures of CDTb, Ib, and PA, and no other open conformation was observed. However, two assumed open-states have been observed in electrophysiological studies in PA[2,3].

1 Hardenbrook, N. J. *et al.* Atomic structures of anthrax toxin protective antigen channels bound to partially unfolded lethal and edema factors. *Nat Commun* **11**, 840, doi:10.1038/s41467-020-14658-6 (2020).

2 Das, D. & Krantz, B. A. Peptide- and proton-driven allosteric clamps catalyze anthrax toxin translocation across membranes. *Proc Natl Acad Sci U S A* **113**, 9611-9616, doi:10.1073/pnas.1600624113 (2016).

3 Yamini, G. *et al.* Hydrophobic Gating and 1/f Noise of the Anthrax Toxin Channel. *J Phys Chem B* **125**, 5466-5478, doi:10.1021/acs.jpcb.0c10490 (2021).

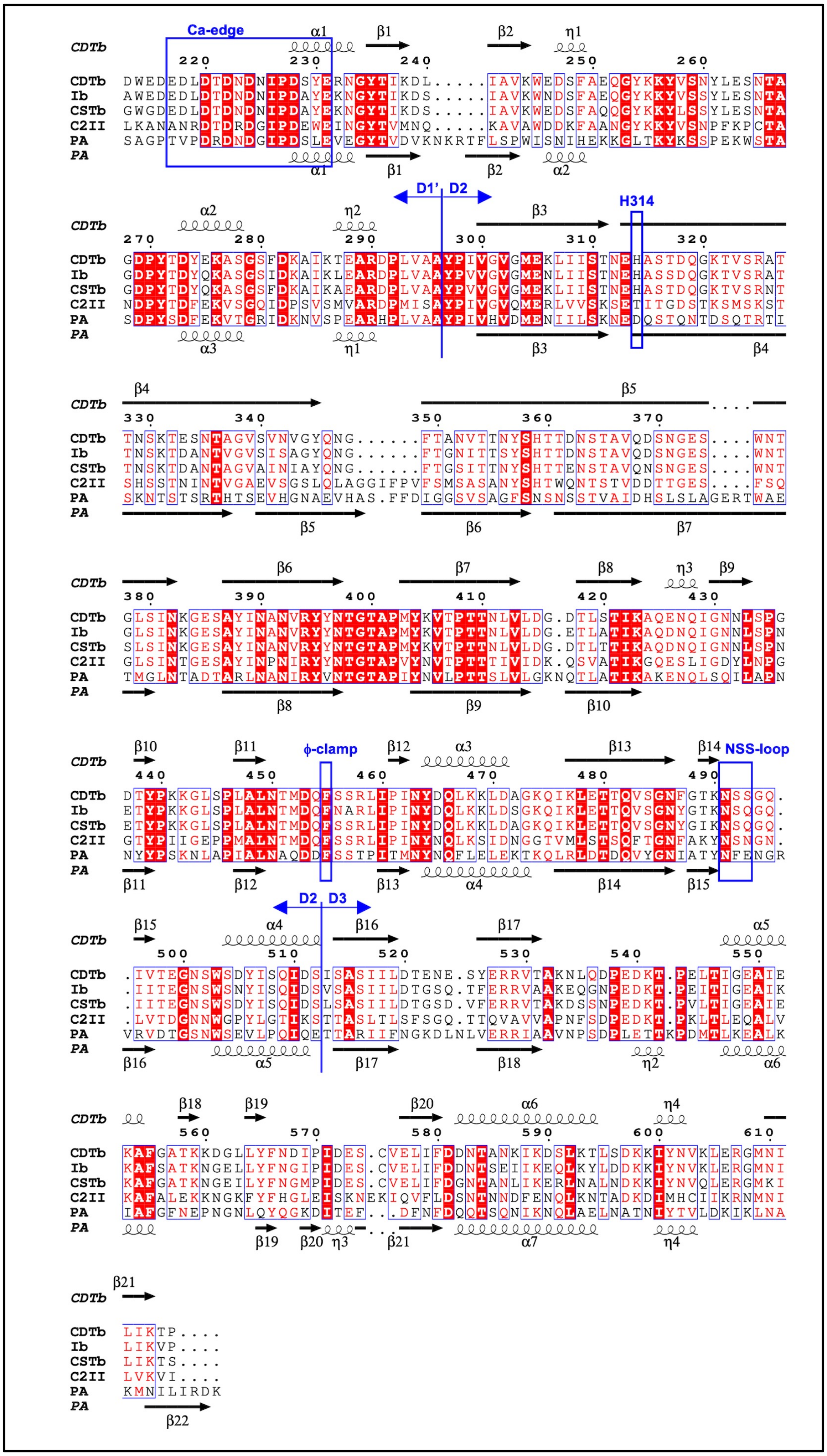

Supplementary Fig. 1: Sequence alignment of binary toxin B-components.
References used for secondary structures are CDTb-pore (PDB ID: 6UWR) and PA-pore (PDB ID: 3J9C). Domain borders and the residues forming constriction sites are indicated. Alignments with secondary structures were made using the ESPript server. (Robert, X. and Gouet, P. (2014) Deciphering key features in protein structures with the new ENDscript server. *Nucl. Acids Res.* **42**(W1), W320-W324 - doi: 10.1093/nar/gku316)

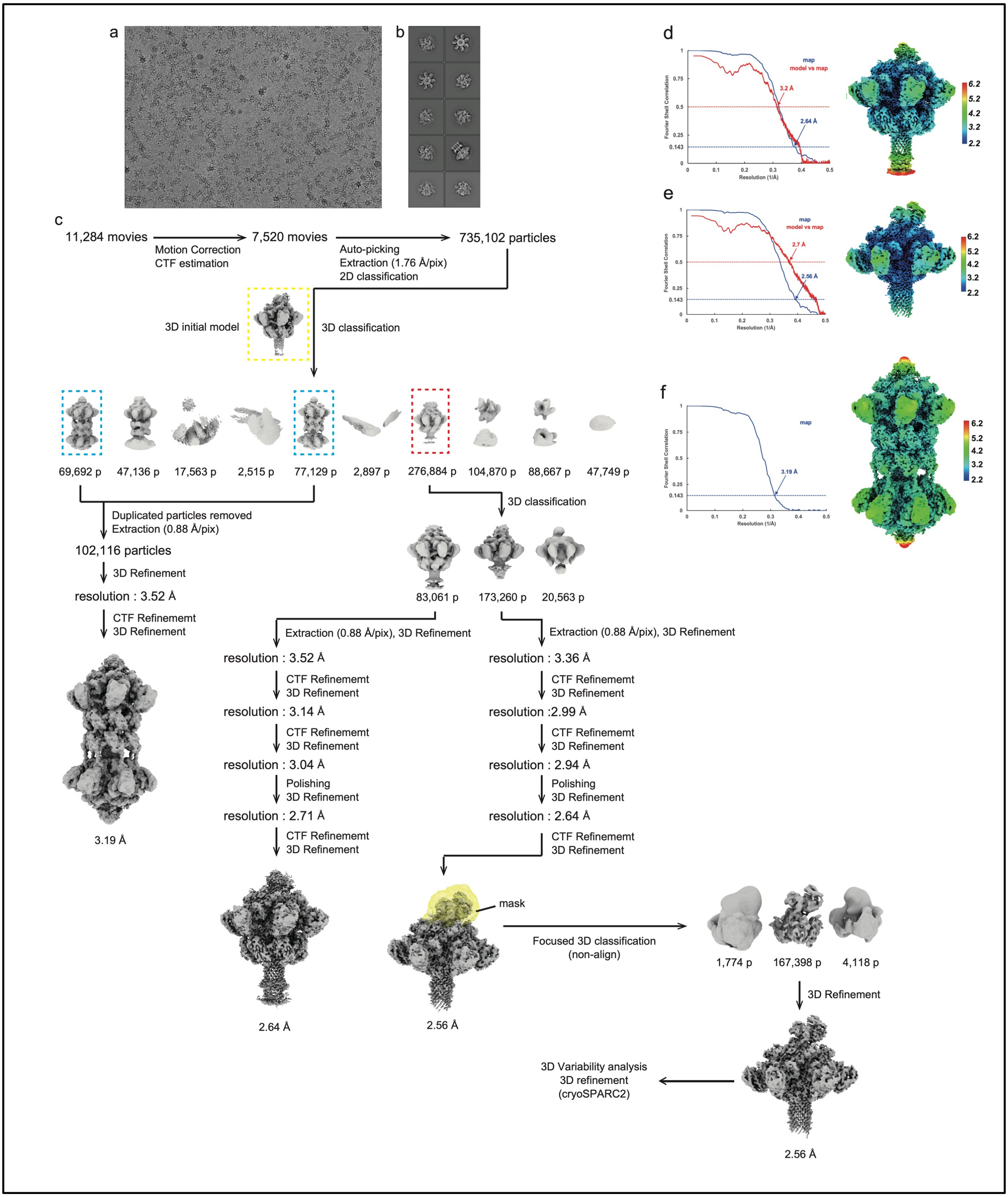

Supplementary Fig. 2: Single-particle analysis of CDTa-bound CDTb-pore.
a, Representative micrograph. **b**, 2D classification. **c**, Flow chart of cryo-EM image processing. The following 3D variability analysis is described in main Fig. 6, results and methods. FSC curves of the final map and final 3D reconstructions of **d,** long CDTa-bound CDTb-pore, **e,** short CDTa-bound CDTb-pore, and **f,** di-heptamer CDTa-bound CDTb-pore colour-coded according to local resolution.

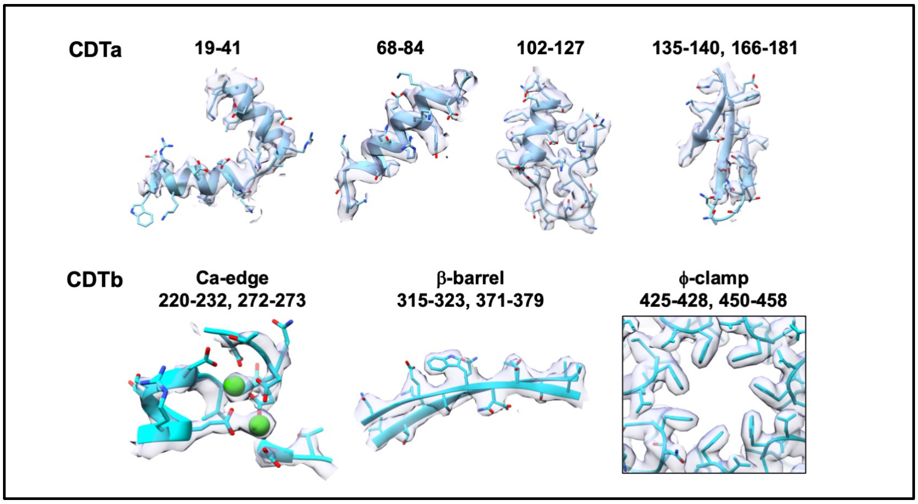

Supplementary Fig. 3: Cryo-EM density maps and models of short CDTa-bound CDTb-pore.

Atomic model with representative maps at 2.56-Å resolution.

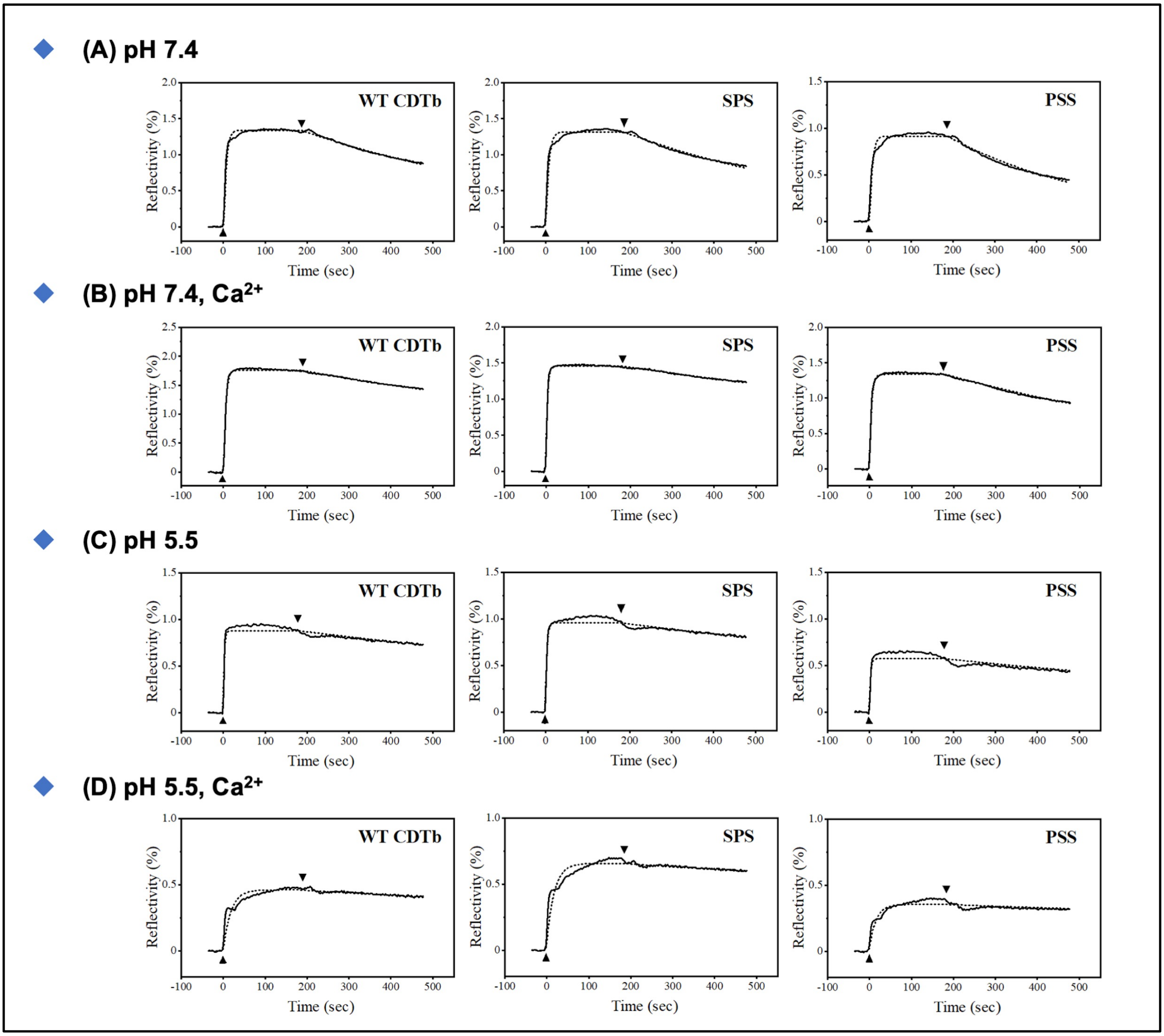

Supplementary Fig. 4: SPR analysis of CDTa binding to CDTb.

CDTb (WT-NSS-loop) and NSS mutants (SPS and PSS) were immobilized using standard amine coupling onto NHS-activated chips. CDTa (1 µM) was injected, and the association was recorded for 180 s. Dissociation was monitored by injecting running buffer alone for 300 s. The triangles indicate the start of association and dissociation, respectively. Fitting curves are shown in dotted lines and the fitting parameters are summarized in Table 2. (A) pH7.4 (B) pH7.4, $CaCl_2$ 1 mM (C) pH 5.5 (D) pH 5.5, $CaCl_2$ 1 mM.

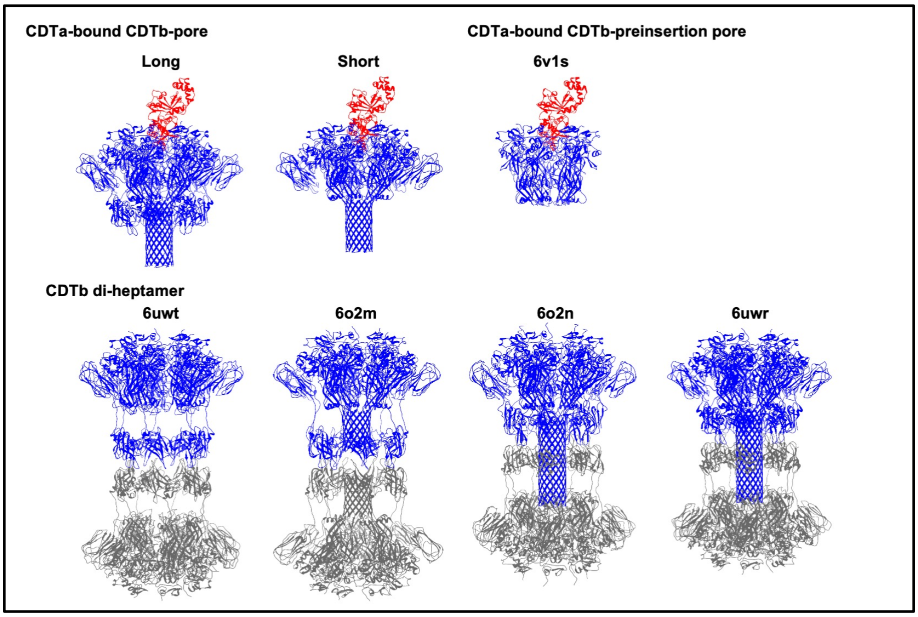

Supplementary Fig 5: Reported structures of CDTb, including CDTa-bound CDTb-pores. CDTa is shown in red and CDTb is shown in blue or grey. CDTa-bound CDTb-pore (long) (PDB ID: 7VNN), CDTa-bound CDTb -pore (short) (PDB ID: 7VNJ), CDTa-bound CDTb-pre-insertion state (PDB ID: 6V1S), di- heptamer (PDB ID: 6UWT, 6O2M, 6O2N, and 6UWR).

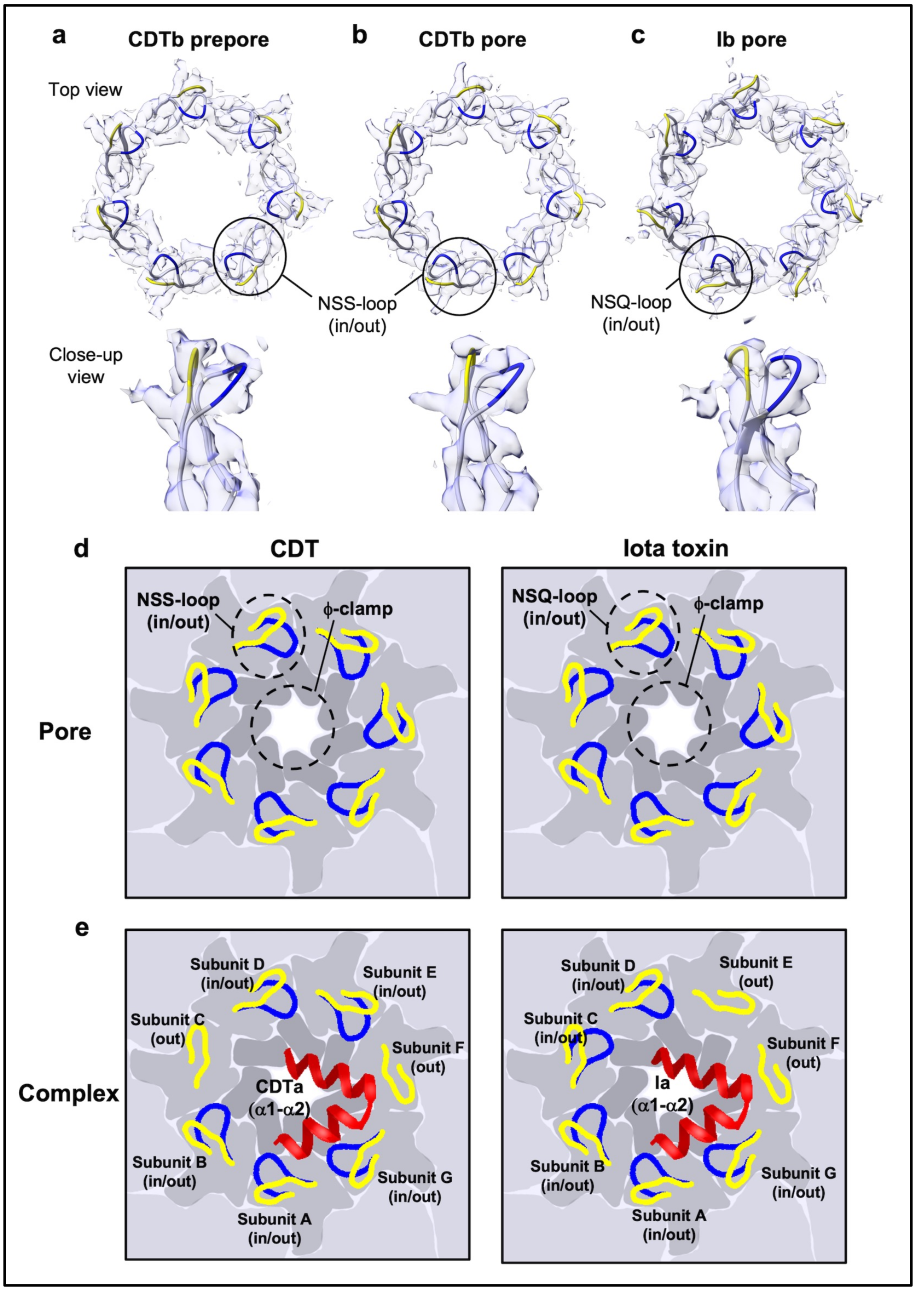

Supplementary Fig. 6: Loops of CDTb and Ib.

CDTb di-heptamer cryo-EM map (EMD-20926) and Ib-pore map (EMD-0721) were visually inspected for conformations of NSX-loops. Each NSS-loop (CDTb) or NSQ-loop (Ib) is shown in blue (in) or yellow (out). **a, b** Transparent CDTb di-heptamer maps fitted to 'in' and 'out' loops. **a** Prepore. **b** Pore. **c** Transparent Ib-pore map was used fitted to 'in' and 'out' loops. **d, e** Schematic of conformational changes in NSS- and NSQ-loops, between apo and A component-bound complex states. **e** Upon A component binding, the NSS-loops of subunit C,F are biased to the 'out' conformation in the CDTb-pore, while the NSQ-loops of subunit E,F are biased to the 'out' conformation in the Ib-pore.

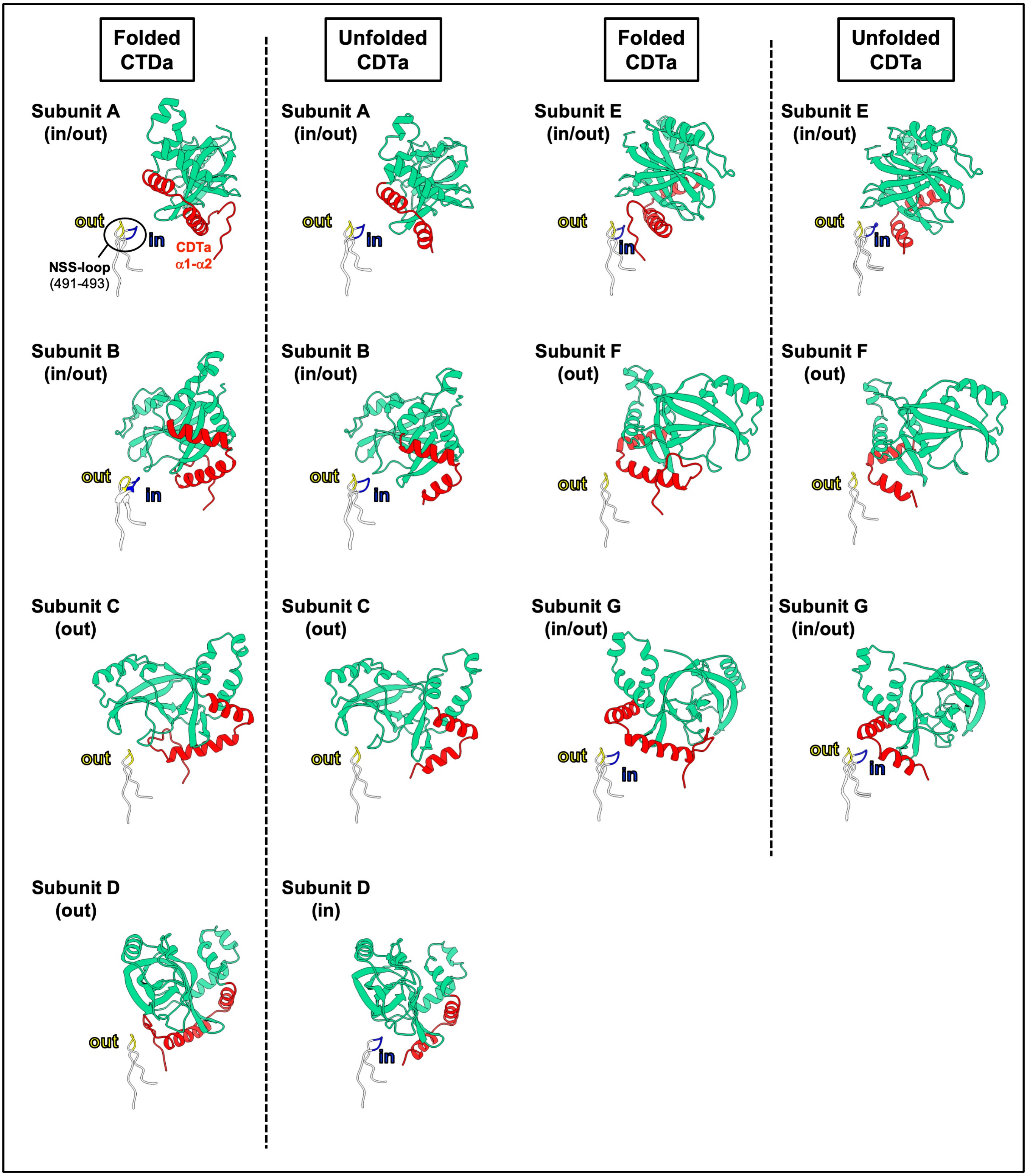

Supplementary Fig. 7a: Side-by-side NSS-loop comparison
Loops are coloured as blue (in) or yellow (out). The CDTa N-terminus is shown in red.

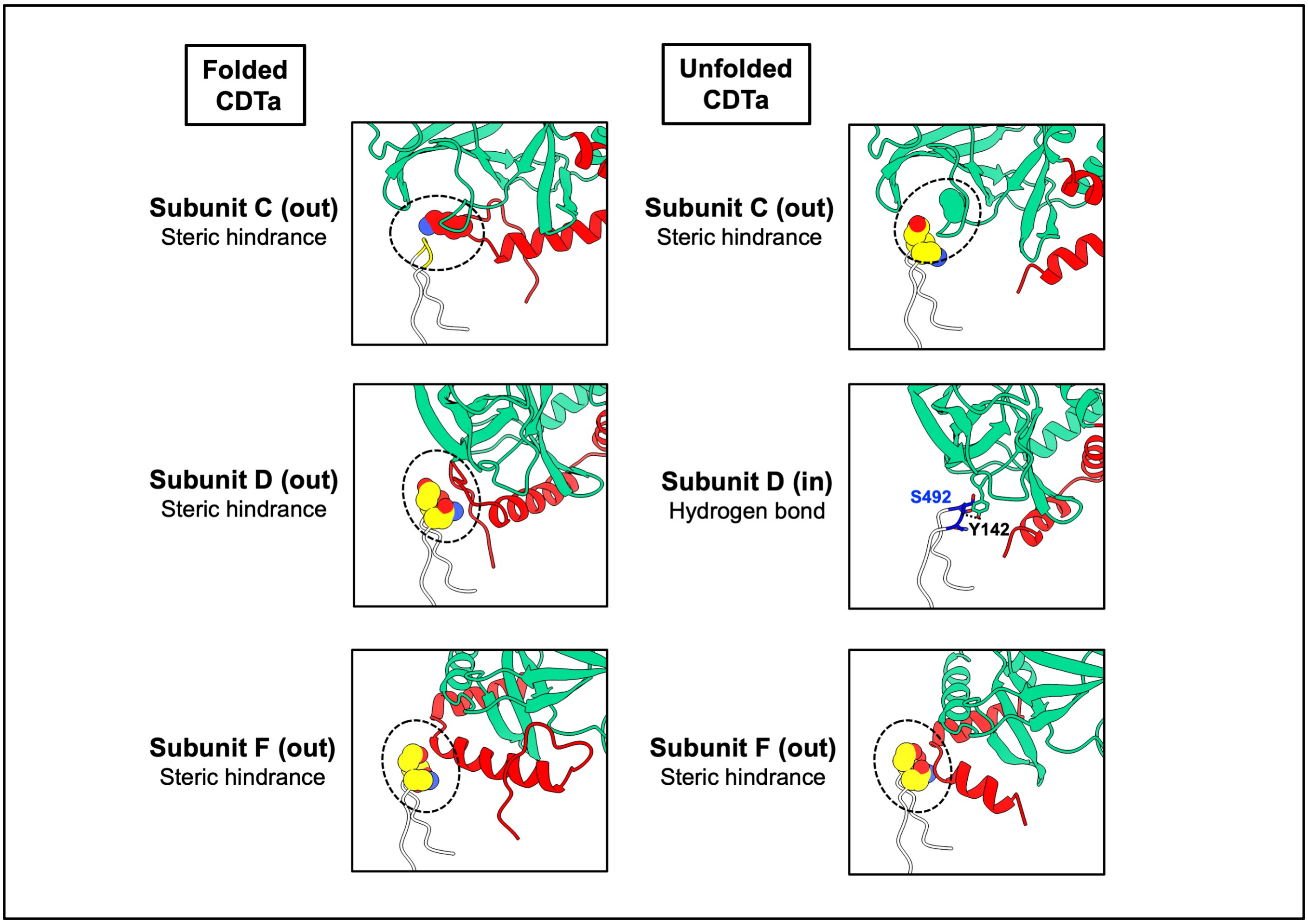

Supplementary Fig. 7b: NSS-loop conformational biases in the contexts of folded vs. unfolded CDTa.

Biases in conformations are caused by steric hindrance with CDTa, shown using sphere models. Biases caused by hydrogen bonding with CDTa is shown as a dotted line.

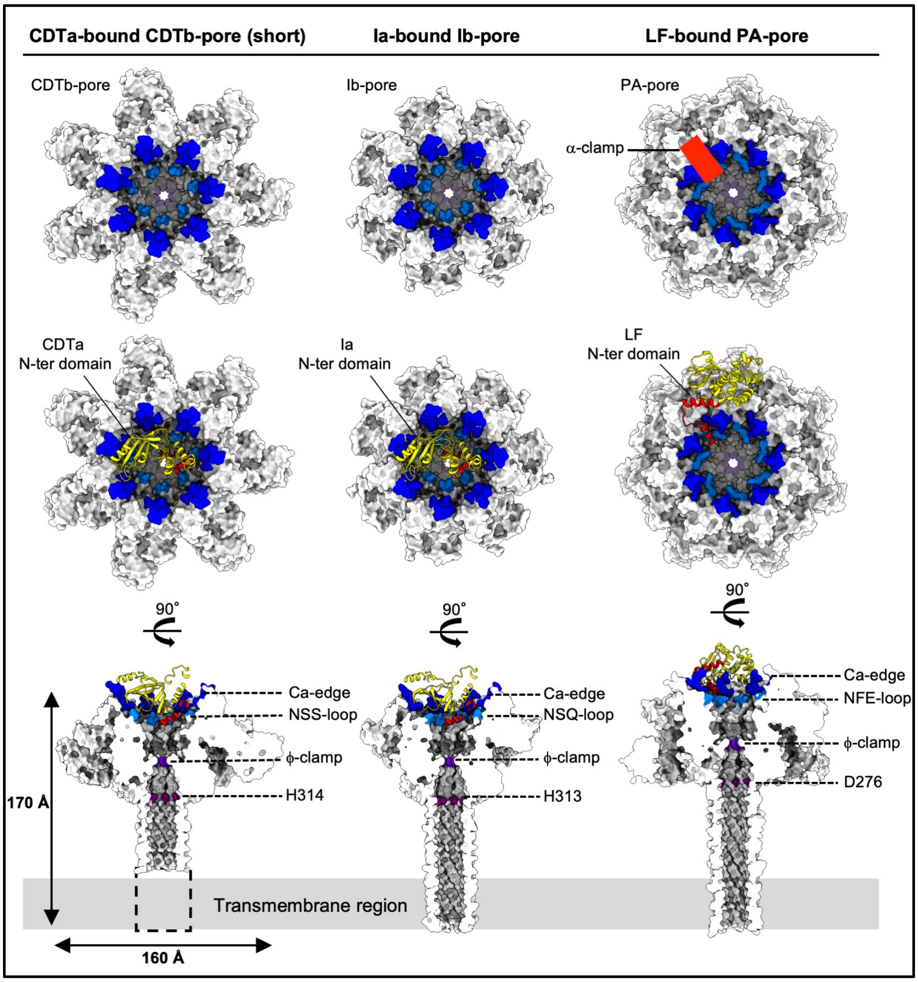

Supplementary Fig. 8: Comparison of CDTb with Ib and PA, shown with α-clamp site. Structures of CDTa-bound CDTb-pore (PDB ID: 7VNJ), Ia-bound Ib-pore (PDB ID: 6KLW) and LF-bound PA-pore (PDB ID: 6PSN) are shown. The constrictions designated Ca-edge, NSS (NSQ, NFE) loops, ϕ-clamp and H314 (H313, D267) are shown in blue, cyan, purple and purplish-red respectively. N-terminal α-helices α1 and α2 are shown in red. C-terminal domains of A-component are hidden to make the pores visible. *Top,* Top views of the pore in the A component-bound state without showing the A component. The α-clamp of the PA-pore, at which the N-terminal α-helix of LF binds, is indicated with a red square. *Middle,* Top views of the A component-bound B component pores. *Bottom*, Cut-away side views of A component-bound B component pores.
