# Supplementary material for: Cryo-EM structures of the translocational binary toxin complex CDTa-bound CDTb-pore from Clostridioides difficile
Source: Nat Commun. 2022 Oct 17;13:6119. doi: 10.1038/s41467-022-33888-4 (PMC9576733; doi:10.1038/s41467-022-33888-4)
Supplement: Supplementary file 2 — Description of Additional Supplementary Files [file 41467_2022_33888_MOESM2_ESM.pdf]

File name: Supplementary Movie 1

Description: Translocational unfolding of CDTa by 3D variability analysis

The initial 20 frame maps (Frame 0~19) which were reconstructed with particles classified by 3D variability analysis, are shown as side view and side cross-section view. The maps of folded CDTa class and unfolded CDTa class are shown with atomic model. The blue NSS-loops indicate ‘in’ state, and the yellow NSS-loops indicate ‘out’ state. The N-terminal α-helix is coloured as red.
